# Evaluation of a Novel Contactless Electrical Impedance Device for Measuring Respiratory and Heart Rates: A Pilot Study

**DOI:** 10.7759/cureus.18622

**Published:** 2021-10-09

**Authors:** Yoshiaki Iwashita, Satoru Nebuya

**Affiliations:** 1 Department of Emergency and Critical Care Medicine, Shimane University, Izumo, JPN; 2 Collaborative Research Programs of Advanced Medical Electromagnetic Engineering, Shimane University, Izumo, JPN

**Keywords:** vital signs monitor, mechanical ventilation, medical engineering, heart rate, respiratory rate, electrical impedance tomography

## Abstract

Although the respiratory rate is an important vital sign, it is rarely recorded in hospitals given the lack of convenient measurement devices. Posh Wellness Laboratory Inc. (Tokyo, Japan) developed a novel contactless card-type respiratory/heart rate monitoring device that measures electrical impedance variations on the human chest. This study was aimed to test and validate the accuracy of the proposed device compared with conventional medical monitors. To evaluate the card-type monitoring device, we compared the measurements from the device with those from the mechanical ventilators and electrocardiogram monitors. Patients who were hospitalized in the Emergency Department of Shimane University Hospital from April 5 to 30, 2021 were included in this study. A card-type sensor was attached to five patients who agreed to participate in this study. Four of the five patients were receiving mechanical ventilation. The respiratory rate error provided by the card-type sensor remained within 15% compared with the measurements of the conventional medical monitor. In contrast, the heart rate counts were largely different from the measurements. Thus, the proposed device can successfully measure the respiratory rate, whereas heart rate measurements require further improvement. Our small, lightweight, radiation-free, and contactless monitoring device can conveniently measure the respiratory rate of patients. With the improvement of measuring the heart rate, we would like to assess a larger number and a wider range of patients.

## Introduction

The respiratory rate is an important vital sign for procedures such as diagnosing the severity of pneumonia and sepsis and predicting acute deterioration of patients in hospital settings [1-4]. Moreover, measuring the respiratory rate may contribute to detecting the exacerbation of coronavirus disease (COVID-19) early. In fact, with the global spread of COVID-19, several patients showing mild symptoms or asymptomatic patients are treated outside medical facilities. Despite receiving healthcare monitoring, some of these patients die without noticing the severity of their condition.

Currently, recording the respiratory rate is frequently missed compared to oxygen saturation monitoring [5-7]. This is probably because no convenient measurement devices exist. Thus, Posh Wellness Laboratory Inc. (Tokyo, Japan) has developed a contactless card-type respiratory/heart rate monitoring device based on electrical impedance tomography. This tomography technique allows measuring body impedance variations by applying an electrical current. Other examples of the application of this technique include body composition analyzers and lung scans [8,9]. In this study, we aimed to validate the accuracy of our device compared with conventional monitoring devices found in hospitals.

## Materials and methods

This was a prospective observational pilot study conducted at the Department of Emergency and Critical Care Medicine, Shimane University Hospital from April 5 to 30, 2021. The inclusion criteria were patients who visited the emergency room and were hospitalized at the Department of Emergency and Critical Care Medicine in Shimane University Hospital. The patient has to be 18 years of age or older with the ability to consent and agree to participate in this study. Since our card-sensor is a prototype, there is only one available device and only one researcher was available for this pilot study. Patients were enrolled only when the researcher was in charge of the Department of Emergency and Critical Care Medicine. Therefore, the possible number of the participation was limited, and the target number of cases was set at five. We excluded patients who had implanted cardiac pacemakers or similar devices such as implantable cardioverter defibrillator and cardiac resynchronization therapy defibrillator. All study participants provided informed consent. This study was approved by the Shimane University Institutional Committee on Ethics (study no. 4994 approved on November 6, 2020).

Study protocol

After the patients who participated in this study arrived at the emergency medical center or intensive care unit, the card-type sensor was placed close to their chest. Each patient was on the bed, and the angle of the bed depended on the usual treatment, either supine or gauge-up. The device software was executed in a dedicated computer to start measuring the impedance variations and calculate the corresponding respiratory and heart rates. The card-type sensor data were recorded every minute. The data from invasive mechanical ventilator and electrocardiogram monitors and corresponding respiratory rate were recorded every five minutes. The data of each patient were recorded for 15 minutes, obtaining three records from the monitors per patient.

Card-type monitoring device

**Figure 1:**
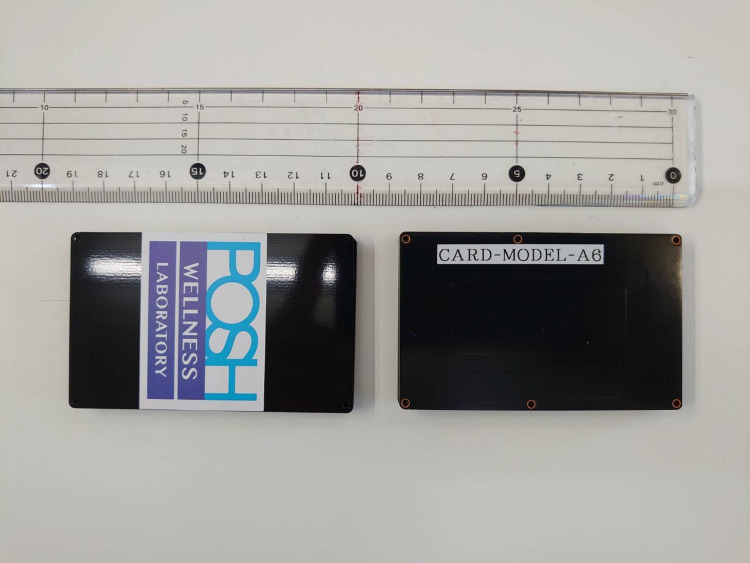
Photograph of the developed card-type monitoring device. This is a photograph of the card-type sensor. The device has a size of 55.0 × 85.6 × 6.9 mm (length × width × height) and weighs 37.0 g.

Posh Wellness Laboratory Inc. fabricated a prototype of a card-type respiratory/heart rate monitoring device. The device has a size of 55.0 × 85.6 × 6.9 mm (length × width × height) and weighs 37.0 g (Figure 1). The device can be inserted into a chest pocket. As hospital gowns have no pockets, we placed the card in a bag and glued it to the gown with a sticker. The device measured the bioelectrical impedance of the lungs and heart. The measured impedance variations were converted into respiratory and heart rates. This can be accomplished in indirect measurement [5]. The impedance data were transmitted to a dedicated computer through a Bluetooth interface. The current model of the sensor takes two hours for charge and the duration for use is six hours when sampling the data every one minute, and 12 hours when sampling the data every two minutes.

Electrocardiogram and mechanical ventilator monitors

IntelliVue electrocardiogram monitors (Philips Healthcare, Amsterdam, Netherlands), as well as Servo-S (Maquet, Rastatt, Germany) and Hamilton-C6 (Hamilton Medical, Bonaduz, Switzerland) mechanical ventilators, were available at Shimane University Hospitals during the study. The measured respiratory rate provided by the mechanical ventilator was used as the reference value in patients receiving mechanical ventilation, while the respiratory rate was manually measured in non-mechanically ventilated patients. The values displayed on the electrocardiogram monitor were also recorded.

Statistical analysis

Descriptive statistics are calculated in R statistical software (R Core Team, Vienna, Austria).

## Results

During the study period, a total of 22 patients were admitted to our department. Due to the availability of the device and researcher, five patients were eligible for this study and agreed to participate. Table 1 lists the patient characteristics.

**Table 1:** Demographic data of the study patients.

Case	Age	Gender	Height (cm)	Weight (kg)	BMI	Diagnosis	Oxygen support	Past medical history
1	26	Female	166	85.7	31.1	Acute intoxication	Mechanical ventilation	Schizophrenia
2	19	Male	167.3	48.2	17.2	Acute intoxication	Mechanical ventilation	Pulmonary hypertension
3	86	Male	167.6	47.5	16.9	Acute subdural hematoma	Mechanical ventilation	Renal failure
4	86	Male	165	58.2	21.3	Hypovolemic shock	Oxygen free	Dementia
5	85	Female	145	61.7	29.3	Sepsis	Mechanical ventilation	Colon cancer

The median age of the patients was 85 years (range, 26-86 years). Three of the five patients were male. Their median height was 166.0 cm (interquartile range, 165.0-167.3 cm), while their median weight was 58.2 kg (interquartile range, 48.2-61.7 kg), and their median body mass index was 21.1 (interquartile range, 18.3-27.4). Four out of the five patients were receiving mechanical ventilation during measurements. Thus, the respiratory rate of the card-type sensor was compared to that provided by the mechanical ventilator. As the remaining patient was not receiving mechanical ventilation, the reference respiratory rate was directly counted by an experimenter. The respiratory rate from the electrocardiogram monitor was also recorded. The heart rate was obtained from both the card-type sensor and electrocardiogram monitor. The measured vital signs are listed in Table 2.

**Table 2:** Measured data on card-type sensor and monitors. Card: card-type sensor; Temp: body temperature; MV: mechanical ventilation; SpO2: oxygen saturation.

Case	Heart rate (card)	Respiratory rate (card)	Heart rate (monitor)	Respiratory rate (measurement)	Respiratory rate (monitor)	Blood pressure	SpO2	Oxygen	Temp (℃)
1	99	19	94	18	18	128/74	100	MV	37
1	98	19	94	18	18	129/77	99	MV	37
1	95	19	93	18	18	128/74	100	MV	37
2	88	16	81	16	17	97/46	95	MV	36.1
2	93	13	89	15	16	74/43	95	MV	36.1
2	105	16	106	15	17	77/49	95	MV	36.1
3	126	17	76	17	17	128/60	100	MV	36.3
3	108	17	79	17	17	124/59	100	MV	36.3
3	132	17	77	17	17	124/58	100	MV	36.3
4	115	14	62	14	14	94/51	98	Free	36.5
4	117	14	67	14	15	101/55	99	Free	36.5
4	124	18	71	16	19	106/57	100	Free	36.5
5	76	16	73	15	15	112/61	99	MV	36
5	77	16	76	15	14	109/58	100	MV	36

**Figure 2:**
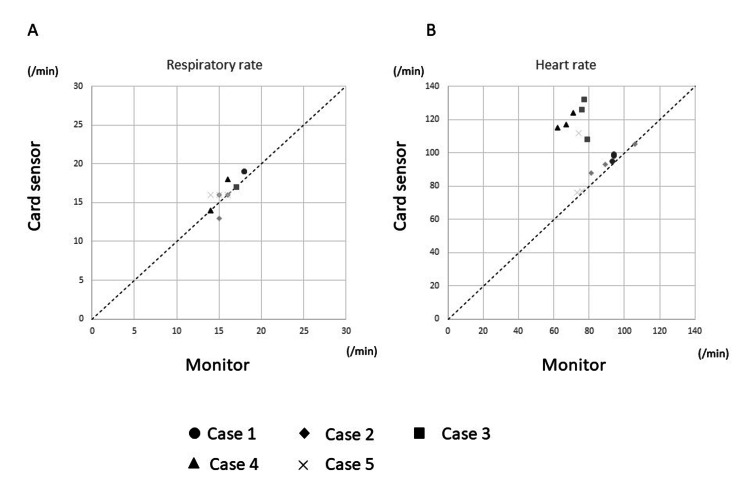
Relations of respiratory and heart rates between measurements from the card-type sensor and corresponding monitor. Dotted line - reference line with x = y.

The median respiratory rate obtained from the mechanical ventilator monitor or direct count was 16 breaths per minute (interquartile range, 15-17.5 breaths per minute), and the median heart rate obtained from the electrocardiogram monitor was 77 beats per minute (interquartile range, 73.5-91.0 beats per minute). The relations between the measurements from the card-type sensor and the corresponding monitor are shown in Figure 2.

## Discussion

We evaluated the proposed card-type respiratory/heart rate monitoring device in hospital settings. For the respiratory rate, the card-type sensor measurements were suitably correlated with the monitor values. The card-type sensor measurement showed a variation within ±15% compared with the monitor measurements. In contrast, for the heart rate, the measurements for all the cases in patients 3 and 4 and for one case in patient 5 seemed to be outliers.

The proposed device successfully measured the respiratory rate in most cases. The respiratory rates from four out of the five patients were compared to those provided by a mechanical ventilator, which counts the respiratory rate directly, showing the high reliability of the proposed device. Therefore, the card-type sensor can be used as a reliable monitor for the respiratory rate.

The importance of measuring the respiratory rate is well-documented in the literature. For instance, it can allow to determine the severity of pneumonia [1] and diagnose sepsis [2]. Various studies have shown that the respiratory rate can be used to predict the initial signs of acute deterioration in hospitalized patients [3,4]. In fact, respiratory and heart rates are the third and fifth most important parameters to predict such deterioration, respectively [7]. Despite its importance, the respiratory rate is rarely recorded in hospitals [8,9]. Although the introduction of a rapid response team and education may promote the recording of the respiratory rate [10,11], missing records persist. The proposed card-type sensor is a small, lightweight, radiation-free, and contactless device that can be used for continuous monitoring of the respiratory rate.

Respiratory rate measurements may also be important in predicting acute exacerbation of COVID-19. Since its emergence, COVID-19 has spread worldwide, affecting millions of people. One of its characteristics is the presence of mild symptoms or asymptomatic progression in many patients. Although most of these patients recover spontaneously, some of them develop an exacerbated condition and even die. Due to the high contagion rate, not all patients can be hospitalized and should stay at home or in accommodation facilities, with some patients suffering from cardiac arrest in prehospital settings [12]. COVID-19 is known to produce intrapulmonary shunting and results in “silent hypoxia” or “happy hypoxia,” that is, hypoxia without dyspnea [13,14]. In addition to oxygen saturation, the respiratory rate may be fundamental to predict mortality in COVID-19 patients [15]. To enable home care, various wearable monitors to measure the heart rate, weight, oxygen saturation, temperature, and blood pressure are available [16,17]. The effectiveness of diagnosis and follow-up of diseases such as heart failure, chronic obstructive pulmonary disease, and sleep apnea has also been reported. However, the respiratory rate in prehospital settings could not be continuously measured. The proposed card-type sensor can be used in non-hospital facilities as a wearable device. Adding the respiratory rate to home monitoring may facilitate the prediction of COVID-19 progression.

Our study has various limitations that remain to be addressed. First, the sample size was small, and the study duration was short. After this pilot study, we plan to conduct a larger study. Second, the developers of the monitoring device acquired the measurements, possibly leading to bias. Further multicenter studies with various experimenters should be conducted. Third, the accuracy of heart rate monitoring was not high. The possible reasons for this were as follows: the sensitivity of the depth was not high and the sensitivity of the counting algorithm was not accurate. Cases 1 and 5 were females with higher BMI and thus higher fat, which enables accurate impedance measurement. Cases 3 and 4 were thin males with lower fat and the air in front of the heart disturbed the measurement. Case 2 was a thin male but he has cardiomegaly due to pulmonary hypertension that made better sensing. Posh Wellness Laboratory is currently updating the device for better sensing of heart rate.

## Conclusions

The proposed card-type monitoring device allows to accurately measure the respiratory rate in a wearable setup. Its measurements are suitably correlated with mechanical ventilator values, but calculation corrections are required to improve the heart rate estimation. A larger study to further verification with heart rate count update is needed. The card-type sensor may facilitate the early detection of respiratory infections in patients receiving home care.
